# Human Clinical Relevance of the Porcine Model of Pseudoallergic Infusion Reactions

**DOI:** 10.3390/biomedicines8040082

**Published:** 2020-04-08

**Authors:** János Szebeni, Raj Bawa

**Affiliations:** 1 Nanomedicine Research and Education Center, Institute of Translational Medicine, Semmelweis University, 1089 Budapest, Hungary; 2 SeroScience Ltd., 1089 Budapest, Hungary; 3 SeroScience International LLC., Cambridge, MA 02138, USA; 4 Department of Nanobiotechnology and Regenerative Medicine, Faculty of Health, Miskolc University, 3515 Miskolc, Hungary; 5 The Pharmaceutical Research Institute, Albany College of Pharmacy and Health Sciences, Albany, NY 12144, USA; bawa@bawabiotech.com; 6 Patent Law Department, Bawa Biotech LLC, Ashburn, VA 20147, USA; 7 Guanine Inc., Rensselaer, NY 12144, USA

**Keywords:** adverse drug reactions, anaphylaxis, anaphylactoid reactions, shock, nanomedicine, nanoparticle, pigs, complement, pulmonary intravascular macrophages

## Abstract

Pigs provide a highly sensitive animal model for pseudoallergic infusion reactions, which are mild-to-severe hypersensitivity reactions (HSRs) that arise following intravenous administration of certain nanoparticulate drugs (nanomedicines) and other macromolecular structures. This model has been used in research for three decades and was also proposed by regulatory bodies for preclinical assessment of the risk of HSRs in the clinical stages of nano-drug development. However, there are views challenging the human relevance of the model and its utility in preclinical safety evaluation of nanomedicines. The argument challenging the model refers to the “global response” of pulmonary intravascular macrophages (PIM cells) in the lung of pigs, preventing the distinction of reactogenic from non-reactogenic particles, therefore overestimating the risk of HSRs relative to its occurrence in the normal human population. The goal of this review is to present the large body of experimental and clinical evidence negating the “global response” claim, while also showing the concordance of symptoms caused by different reactogenic nanoparticles in pigs and hypersensitive man. Contrary to the model’s demotion, we propose that the above features, together with the high reproducibility of quantifiable physiological endpoints, validate the porcine “complement activation-related pseudoallergy” (CARPA) model for safety evaluations. However, it needs to be kept in mind that the model is a disease model in the context of hypersensitivity to certain nanomedicines. Rather than toxicity screening, its main purpose is specific identification of HSR hazard, also enabling studies on the mechanism and mitigation of potentially serious HSRs.

## 1. Introduction

Infusion reactions, i.e., acute hypersensitivity reactions (HSRs) induced by intravenously (i.v.) administered drugs and certain other compounds represent an old, yet unsolved immune barrier to the clinical use of numerous nanomedicines, radiologic contrast agents, biologicals, enzymes, muscle relaxants, and a variety of other pharmaceutical products [1,2,3,4,5,6,7]. Although the standard, empiric preventive measures effectively attenuate these adverse drug reactions (ADRs) in most cases [3,4], there has been no breakthrough in the prediction and prevention of occasional Grade IV–V severe adverse reactions (SARs), also known as severe adverse events (SAEs), culminating in anaphylactic (or anaphylactoid) shock or death [3,8,9,10,11,12,13]. Such SARs may not only preclude the patient from treatment with a potentially life-saving drug, but their clustering may entail the suspension or withdrawal of the drug from clinical use, thereby having negative implications for drug manufacturers as well. These facts lend substantial importance to better understanding the mechanism of drug-induced HSRs, which can be perceived as “stress reactions” in blood along the innate immune-circulatory system axis [14].

Since HSRs cannot be reproduced in vitro, research and development in this field necessitates the use of animal models. One such model is the so-called porcine complement (C) activation-related pseudoallergy (CARPA) model [15,16,17,18] which involves i.v. injection of the test drug(s) into pigs. In the case of immune reactivity, the drug administration then triggers more or less severe cardiopulmonary, hemodynamic, hematological, skin, and laboratory changes similar to those observed in patients displaying HSRs to a variety of drugs and agents [15,16,17,18]. It is this concordance of symptoms that provides a rationale for the use of pigs to model human HSRs. The name “CARPA” derives from a large body of experimental evidence for C activation playing a causal or contributing role in the reactions (Table 1). However, it is critical to emphasize that C activation is not the only mechanism of these reactions. The mechanism of HSRs is complex and varies in different species under different conditions, also involving C-independent pathways, referred to as C-independent pseudoallergy (CIPA) [19,20].

Table 1 lists 30 experimental studies [15,21,22,23,24,25,26,27,28,29,30,31,32,33,34,35,36,37,38,39,40,41,42,43,44,45,46,47,48,49] which utilized the pig model to analyze the cardiopulmonary adverse effects of different nanoparticles (NPs) or other agents. Some of these studies highlighted the concordance of HSR symptoms in pigs and hypersensitive patients [25,26,27,28,29,32,33,46,47,48], others addressed the mechanism of HSRs [15,21,22,23,24,25,26,27,28,29,30,31,32,33,36,41,42,43,44,45,46,47], and yet others focused on the prevention of HSRs by pharmacological intervention [15], or by optimizing the structure [24,44] or administration protocol [24,37,48] of NPs. Importantly, many of these studies were initiated mainly for preclinical safety evaluation of nanomedicines [15,22,23,30,31,35,37,39,40,41,42,43,46,49], a term interchangeably applied for “nanoparticulate drugs” or “nanopharmaceuticals”, or “drug carrier nanosystems”.

## 2. Challenge to the Pig Model’s Human Relevance and Utility in Preclinical Safety Assessment

Despite the established use of pigs to study NP-induced cardiopulmonary distress for three decades (Table 1), a recent review questioned the suitability of the model for nanomedicine safety assessment, vociferously arguing against its use [50]. This review claimed that nanomedicine safety assessment in the porcine model might be “inappropriate, misleading, scientifically questionable”, and it warned against “advertent promotion and exaggeration” of the model. This evaluation was repeated and extended further in another recent review [51] with the baseless statement that “compulsory nanomedicine response tests in pigs should not be advertently promoted, and imposed on pharmaceutical industry”. The reason provided for these broad assertions in the two reviews is that the pulmonary response to NPs is a “global” phenomenon wherein a population of pulmonary intravascular macrophages (PIMs) indiscriminately respond to NPs with the secretion of thromboxane A2 (TXA2), the classic mediator of cardiopulmonary distress. Thus, -the authors argued- the porcine test “excludes otherwise promising nanopharmaceuticals from clinical development on safety grounds that are not relevant to wider human populations” [50,51].

Given the public focus on the safety of nanomedicines and the fundamental need for an animal model to study infusion-related HSRs, consideration of all information on the different models is important. As for the pig model, in fact, the discordance of HSR frequency to certain nanomedicines between humans and pigs (i.e., roughly 2-10% in man while near 100 % in pigs) has always been a contentious issue, dividing the judgment on the model’s human relevance. Accordingly, the aim of this review is to provide an update regarding the *pros* and *cons* of the pig model [18] while addressing the issues raised in the referred critical reviews [50,51]. Moreover, this review will highlight the CARPA model’s increasing recognition and deployment.

## 3. Scrutiny of the Challenge to the Pig Model: Facts and Questionable Conclusions

The referenced critical reviews [50,51] contain experimentally established facts as well as conclusions that argue against the utility of the porcine CARPA model. For a systematic analysis and clarity, Table 2 separates the facts and claims against the model that we find arguable, along with giving some annotations (italicized text) where necessary for better understanding.

### 3.1. Gaps in the Theory Attributing HSRs to Robust Phagocytosis of NPs by PIM Cells

The first arguable point (Claim 4) in the critique of the pig model is that the HSRs to NPs in this species is a “global” phenomenon due to the robust, non-specific phagocytosis of NPs by PIM cells in the pulmonary circulation of pigs and other cloven-hoof species [44,50,51,59]. Specifically, the mechanism was suggested to involve a C-independent “transient link” between phagocytosis and TXA2 secretion by PIMs which cannot differentiate between reactive and non-reactive NPs [44]. However, in lack of dedicated studies on the role of phagocytosis in TXA2 secretion, the experimental foundation of this proposal is unclear. In fact, such a hypothesis is inconsistent with the known enhancement of phagocytosis by surface-bound C3b and its derivatives, and also with a long list of observations on significant C-dependence of HSRs to NPs in both humans and pigs [19,45] (Table 1). Even the reaction to PS-NPs in pigs, which was claimed as being C-independent [44], turned out to involve C activation-related opsonization [45]. Hence, contrary to the dismissal of CARPA [44,59], the PIM response to PS-NPs also represents CARPA, at least in part. On the other hand, the really C-independent IgG Fcγ-receptor-mediated anaphylactic pathway [59] would also have specificity to the reactogenic drug or agent, dictated by the Fab of IgG.

Another problem with the C-independent phagocytosis-TXA2-link hypothesis is the time course discrepancy between phagocytosis, TXA2 release, and pulmonary reactions in pigs. The recent review [51] argues that the time course of phagocytosis coincides with the peak of TXB2 release, and, hence, pulmonary response of pigs, while such coincidence with C activation is not present in a pig whole blood assay in vitro [44]. Specifically, the HSR in pigs starts already at 40–50 s after the injection of PS-NPs and reaches plateau at 1–3 min [15,61], which is paralleled by the time course of PS-NP clearance from pig blood in vivo [44]. On the other hand, C activation by the same NPs in the whole blood assay is absent, or seen only after 5–10 min incubation [44]. However, we would point out several shortcomings of these arguments. First, the evidence of phagocytosis is the visualization of NPs inside macrophages, and the earliest examination performed to establish phagocytosis was performed at 20 min post-treatment [53], which has no relevance to events within 2 min. Second, phagocytosis cannot be equalized with NP capture, as the observed rapid clearance of NPs from blood, which strongly correlates with HSRs, may be a consequence of the binding of NPs to PIMs and other cells without ongoing phagocytosis. Third, there is flow cytometric and Western blot evidence for C3 cleavage and C5b-9 deposition on PS-NPs, i.e., C activation, already at 1–2 min [45], while the validity of the whole blood assay showing no C activation was questioned on technical grounds [19]. Finally, the critical authors themselves judged it inappropriate to extrapolate from in vitro C activation data to HSRs in vivo [51].

**Figure 1:**
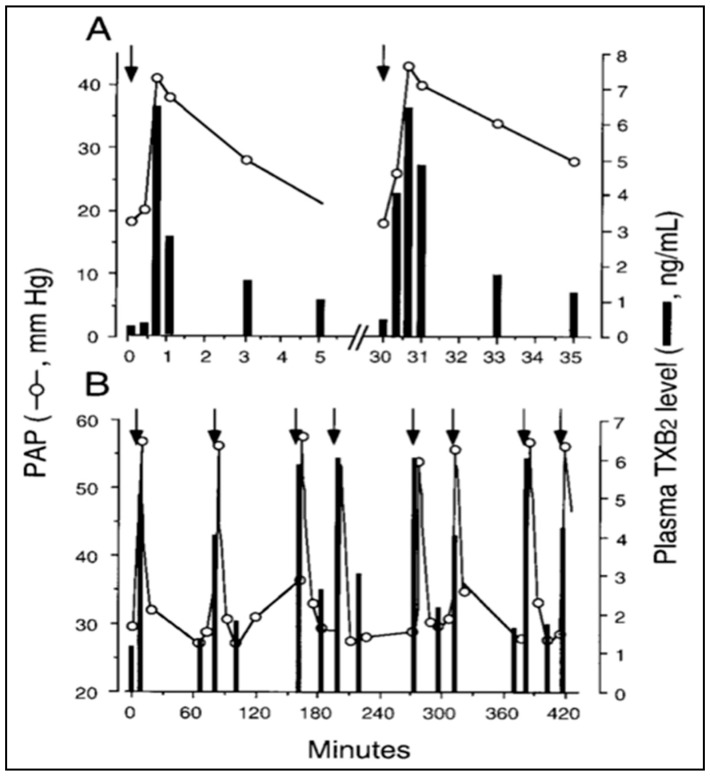
Time course of liposome-induced changes in plasma TXB2 and PAP in pigs. Two animals were repetitively injected with liposome boluses, and changes in PAP (circles) and plasma TXB2 (bars) were plotted as a function of time for the first two injections in one pig (**A**) or over 7 h in another pig (**B**). Other details are in Ref. [15], from where this figure was reproduced with permission. Arrows here indicate the timing of liposome injection.

Further scrutiny of the time course argument, stating that “robust” phagocytosis of NPs coincides with the HSRs, brings up yet another time-related discrepancy. Namely, the detection of a rise of TXB2 must be preceded by its conversion from TXA2; thus, if TXA2 release is indeed “transiently linked” to robust phagocytosis [44], massive amounts of NPs have to be taken up by PIM cells within 40–50 s. In addition, the plasma levels of TXB2 in pigs displayed an array of peaks closely paralleling the peaks of PAP following repetitive injection of the same liposomes within 30 min or over 7 h (Figure 1A,B) [15]. Thus, if the explanation for the pulsatile release of TXA2 boluses in blood is phagocytosis or any endocytosis-involving process, it implies not only an instant maximal engulfment of NPs at the first time, but capability for identical “bites” many times on the minute time scale, over hours (Figure 1A,B, respectively). These experimental observations are difficult to reconcile with textbook information on phagocytosis that describes it as a gradual, unidirectional, saturable process requiring receptor binding, and phagosome internalization with rearrangement of the cytoskeleton. It seems to be hardly linkable with pulsating release of TXA2 [15].

Taking these facts and considerations *in toto*, we suggest that the rapid clearance of PS-NPs and other reactogenic NPs from blood reflects rapid binding to PIM and other cell surfaces, and the instant C-independent liberation of TXA2 may be due to increased arachidonate metabolism at the cell membrane level. Details of this “second hit” on allergy-mediating secretory cells and the exact molecular mechanism of TXA2 release need to be clarified in the future.

Another inaccuracy as listed in Claim 4 of Table 2 is the reference to PIM cells as sole source of TXA2. Macrophages are not the only possible source of TXA2 in pigs and other cloven-hoof animals undergoing HSRs. In addition to mast cells, that are key players in allergy, platelets, polymorphonuclear neutrophils (PMNs), and endothelial cells have all been shown to spill TXA2 in response to NP exposure in blood [53,62,63,64]. Complement activation as a trigger mechanism for these secretory responses by these cells was shown in sheep in the late 1980s [53], providing the earliest proof to the long list of evidence for the validity of the CARPA concept (Table 1).

It should be noted that regarding the source of TXA2 in HSRs the experiment in Figure 1 allows for calculating the total amount of TXB2 released in blood at each liposome exposure. The experiment in Fig. 1, using 20–25 kg pigs, showed tens of micrograms of TXB2 released at each liposome injection, altogether >100 microgram over hours. Assuming that the total number of PIM cells in the lung of an adolescent pig is in the order of 10^8^–10^9^ [56], it would be important to find out whether it is possible that most, if not all, TXA2 could derive from PIM cells.

**Figure 2:**
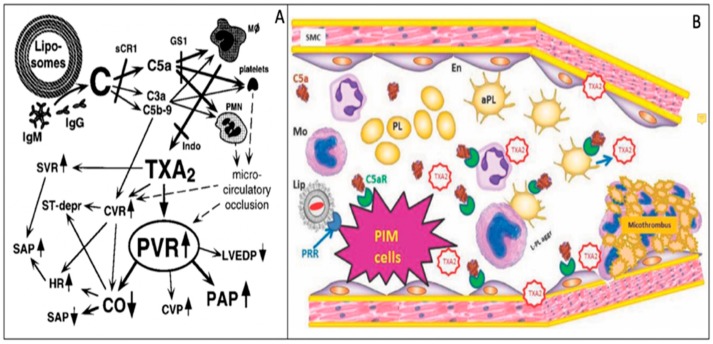
Complex mechanism of liposome-induced CARPA in pigs; schematic (**A**) and visual (**B**) illustration of causally related events, reproduced from Refs. [15] and [66], respectively. (**A**) The arrows indicate causal relationships among the physiological changes; solid and dashed lines indicate experimentally established and hypothetical changes. (**B**) Imaginary snapshot of a pulmonary capillary during CARPA in pigs; the PIM’s TXA2 response to C5a and liposome binding is combined with microthrombus formation on the capillary wall, amplifying the vasoconstrictive effect of TXA2. Abbreviations: (**A**) C, complement; HR, heart rate; Mf, macrophage; Indo, indomethacin; CVR, coronary vascular resistance; ST-depr, ST-segment depression on the ECG; sCR1, soluble C receptor type 1, a C inhibitor; GS1, anti-porcine C5a antibody, PVR, pulmonary vascular resistance, CVR, central vascular resistance, CO, cardiac output, SVR, systemic vascular resistance, HR, heart rate, SAP, systemic arterial pressure, PAP, pulmonary arterial pressure; (**B**) Lip, liposome, aPL, activated platelet; Mo, monocyte; L-P aggr, leukocyte-platelet aggregate; PRR, pattern recognition receptors; En, endothelial cells; SMC, smooth muscle cells.

As a final challenge to Claim 4 in Table 2, focusing solely on PIM phagocytosis/TXA2 release, represents an over-simplification of the mechanism of HSRs. Vasoconstriction by TXA2 is only one pathway in the complex molecular and cellular changes that underlie HSRs (Figure 2A). Specifically, C activation-related activation of anaphylatoxin-receptor positive blood cells entail white blood cell (WBC)–platelet aggregation with subsequent sequestration of micro-emboli in the pulmonary capillary bed [65]. Together with locally formed micro-thrombi and consequent oxidative endothelial damage, these changes act in parallel or in synergy with the vasoconstrictive effect of TXA2 in causing pulmonary blockage of blood flow [15] (Figure 2A,B). Thrombocytopenia and leukopenia with or without secondary leukocytosis are common symptoms of HSRs, reflecting these cells’ direct activation by anaphylatoxins or other stimuli. Thus, whenever these symptoms are present in pigs or other models, a role of TXA2-independent platelet, WBC and endothelial cells activation is likely to be involved.

Moghimi et al. [51] referred to the significant inhibition of HSRs by macrophage depletion by pretreatment of pigs with clodronate-liposomes [44] as a further evidence for the key role of PIMs in HSR reactions. However, the study [44] gives no information on HSRs to these liposomes, although other bisphosphonate liposomes were reported to cause mild HSRs in pigs [30]. If the repeated treatment of pigs with clodronate liposomes [44] also caused mild, or even subclinical HSRs, desensitization may also explain the reduction of HSR, just as empty PEGylated liposomes (Doxebo) desensitizes pigs against Doxil reactions [33]. In addition, clodronate has other effects that also explain the inhibition of TXA2 and pulmonary response. Upon reviewing the literature for such a possible effect we found that clodronate liposomes can reduce the clustering and accumulation of PMN in inflammatory lung and kidney diseases [67,68]. Since an inflammatory cell reaction is likely to contribute to the cardiopulmonary distress in porcine HSRs (Figure 2) [15,66], PMN-inhibition could also contribute to the HSR-reducing effect of clodronate liposomes [44]. Another open question relates to the observation that, despite the total absence of TXA2 response, the pulmonary hypertensive response was not completely abolished by clodronate liposomes [44]. The remaining 50% rise of PAP is not negligible, for example the pulmonary hypertensive effect of Doxebo is similar [33]. Therefore, partial inhibition of PAP at a time of total inhibition of TXA2 response may reflect the involvement of a TXA2-independent reaction pathway, another experimental evidence against the linking of HSRs solely to TXA2 release as a reason for disqualifying the pig model [44,59].

In summary, the key role of PIM cells in nanomedicine-induced HSRs in pigs is undisputed, but referring to these cells’ capability for robust phagocytosis with transiently linked TXA2 secretion as a cause for the sweeping disqualification of the pig model for safety testing is unjustified based on experimentally-derived evidence. The issue should remain open for further scientific analysis and discussion.

### 3.2. The Cardiopulmonary Response of Pigs to NPs Is Not Global

If the phrase “global response” implies that the cardiopulmonary reaction of pigs to nanoparticles is common, general, universal, ubiquitous, omnipresent, uniform or indiscriminate, which are synonyms of “global”, then this claim goes against a large body of scientific evidence showing exactly the opposite. Namely, all previous studies using the model (Table 1) presented quantitative differences among the reactivities of different nanoparticles and controls. In addition, many studies in Table 1 attest to the dose dependence and reproducibility of the response, although different endpoints (i.e., the SAP, HR, blood cell changes, plasma TXB2, and SC5b-9) show more or less individual variation. It is also important to note that there is a phase in porcine HSRs when the animal’s cardiopulmonary response is insensitive to dose escalation, namely, during the state of tachyphylaxis or self-induced tolerance [33]. This phenomenon has been seen in the case of PEGylated liposomes, whereupon the first reactogenic drug dose desensitized the animals for the next and subsequent challenges [33].

**Figure 3:**
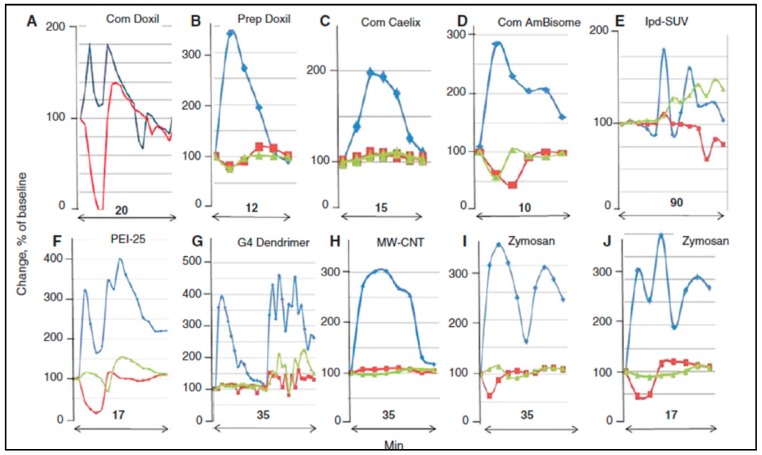
Variation of PAP and SAP waveforms. Panels (**A**–**J**) represent reactions to identical or different NPs, selected from different experiments, wherein the CARPAgenic potential of nanoparticulate drugs or drug carriers were tested in pigs. Minutes indicate the timespan of reactions. Blue, red, and green are PAP, SAP, and heart rate curves, respectively. Changes are shown in percent of baseline. Abbreviations (only here): com, commercial; prep, self-prepared; lpd, lipophilic prodrug-containing liposomes; PEI25, 25 kD pegylated poly(ethylene imine); G4 dendrimer, 4th generation dendrimer; MW-CNT, multiwall carbon nanotube. Reproduced from Ref. [17].

As for the specificity of the pig model, Figure 3 shows that the timing of the up-and-down deflections and wave forms of PAP, SAP, and HR curves substantially differ among NPs under different experimental conditions. On the other hand, the wave peaks and forms are very consistent among different animals for the same nanoparticle trigger under similar experimental conditions.

**Figure 4:**
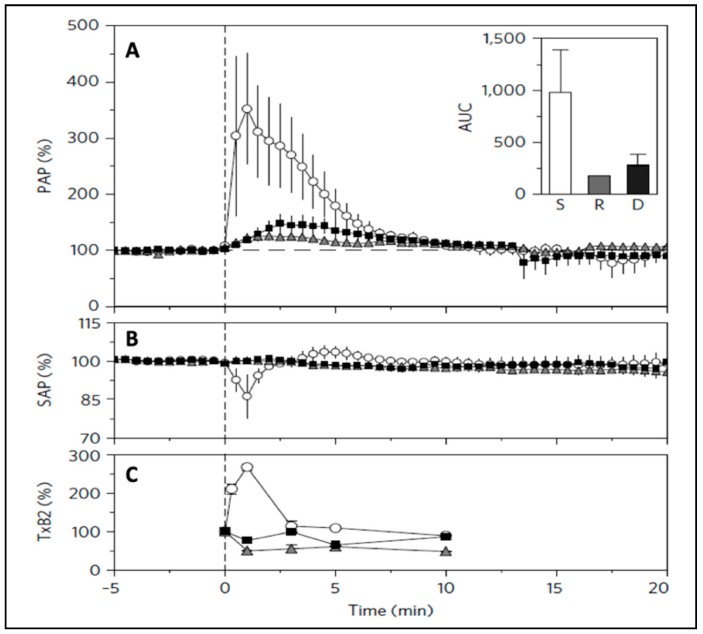
Changes of hemodynamic parameters in pigs after i.v. injection of polystyrene nanoparticles of different shape: spheres (circles), rods (triangles), and disks (squares). Time-dependent changes in pulmonary arterial pressure (PAP) (**A**), systemic arterial pressure (SAP) (**B**), and thromboxane B2 (TxB2) (**C**) following particle injection compared with background (resting phase, before 0 min). injection compared with background (resting phase, before 0 min). Particles (on an equivalent surface area of ~114,300 mm^2^ per 20 kg body weight) were injected at 0 min. Inset: integrated area under the curve (AUC) of the changes in PAP during the first 10 min of injection. d, the results from pig experiments are expressed as mean ± SEM (n = 3). Reproduced from Ref. [44] with permission.

Finally, regarding Claim 5, it should be pointed out that the mentioned study using PS-NPs [44] used three animals in each treatment group to conclude that the cardiopulmonary distress can differentiate among the reactogenicities of 500 nm PS-NPs based on their physical shape (Figure 4). This means astonishingly reproducible spatial resolution of nanoparticle surface curvature, an unsurpassable evidence *against* nonspecific, nonquantitative global response. Taken together, referring to the hemodynamic response of pigs to NPs as global contradicts all experimental evidence, including those published by the main critical author in a top-tier journal [44].

### 3.3. The Issue of Discordant Prevalence of HSRs in Pigs and Humans

Regarding claim 6 in Table 2, namely that the discordant prevalence of HSRs in humans and pigs makes the pig model irrelevant to most humans, and, hence, it wrongly excludes otherwise promising nanopharmaceuticals from clinical development, the first question to ask is: Does this difference in HSRs rates really render the pig model irrelevant to humans?

The answer to this question lies in the use and goal of the pig CARPA assay. In this context, it is important to consider that the model has many features that distinguish it from the standard toxicity tests. Notably, the CARPA test protocol applies the test drugs in bolus form at 2–3 orders of magnitude lower dose than the drug’s planned or established therapeutic dose, thus mimicking the rise of HSRs in man shortly after starting the drug’s infusion, when only a small portion of the drug has reached the blood. Another major difference relative to standard toxicity protocols is that the spectrum of monitored endpoints in the pig model is limited to cardiopulmonary, hemodynamic, blood cell, skin, and some plasma immune mediator changes, all reflecting allergy-related adverse phenomena. In contrast, standard toxicity models explore a great number of organ and body parameters in search for unforeseen abnormalities. Hence, such studies are performed in healthy animals, using a rodent and a large animal species, and the drugs are tested at their therapeutic level and above, in keeping with the human administration protocol for therapeutic or diagnostic application. These differences in methodology reflect the repeatedly emphasized fact that the porcine CARPA model is a disease model, i.e., that of hypersensitivity to nanomedicines [6,16,17,18,19,45,47,48]. Its use in this context is hazard identification and risk assessment for this kind of HSR, and not as a standard toxicology model. To illustrate that the reproducible hypersensitivity of pigs to certain NPs is an advantage rather than a problem, a good example is the discussed study on PS-NP-induced HSRs in pigs, that led the authors to propose a new approach to prevent HSRs, obviously not only in pigs [44]. If the model would truly reflect the prevalence of human HSRs to nanoparticles (2–10%), a minimum of 90–450 pigs should have been used for the study (instead of nine) to allow for the conclusions made, but preferably three-times these numbers to provide statistical power.

Yet another note regarding the prevalence issue, a short editorial by Skotland [60] has been referred to by the critical authors as additional evidence for misusing the pig model. It warns against “trouble” upon performing safety studies by intravenous injection of microparticles in cloven-hoof animals, such as pigs, on the basis of anaphylactic reactions to the ultrasound contrast agent, Albunex, observed in the 1980s. The vivid memory of deadly reactions confirms the timelessness of the problem, and the author added to his “good advice” that the warning against the pig model did not apply if there was “specific reason” for using it. Indeed, there could have been good reason for using the model, to forecast those severe HSRs that have been observed with Albunex, beside the thousands of trouble-free administrations. Albunex was discontinued after the introduction of more effective microbubble-based contrast agents, but the public information still available on the drug’s side effects [69] warns against severe acute allergic reactions requiring emergency measures, and lists dyspnea, arrhythmia, chest pain, swelling of the face, lips, tongue, fever, light-headedness, anxiety, confusion, and sweating among the symptoms, which are also characteristic symptoms of infusion reactions [1,2,3,4,5,6,7,8,9,10,11,12,13]. As more evidence of Albunex’s cardiopulmonary reactivity, it was reported to trigger a biphasic pulmonary response in a subgroup of cardiac patients withdrawn from anti-inflammatory medication [70]. The next-generation ultrasound contrast agents (SonoVue, Optison, and deFinity) continued to cause severe HSRs that led to their temporary or final suspension [71,72,73,74,75,76,77,78,79]. This reactogenicity can be modeled in pigs just like the reactogenicity of the drugs listed in Table 1 (unpublished data).

In summary, taking the discordant prevalence of HSRs in pigs and healthy man as argument against the pig model implies its perception as a standard toxicity, rather than a disease model. It shows misunderstanding of the model’s purpose and utility, despite many previous, strongly emphasized clarifications [6,16,17,18,19,45,47,48]. To reiterate the message in simple words, the pig model is recommended to explore if a hypersensitive individual would become symptomatic to a subtherapeutic dose of the tested drug. The question therefore is not the prevalence of HSR to that drug in the general population but the risk of HSRs to a subtherapeutic dose in the rare cases of hypersensitive patients. Because SAEs even in a small fraction of patents represents a major health and economic problem, contraindicating the porcine assay excludes the identification of nanomedicines that can potentially cause such SAEs.

### 3.4. The Pig Test Can Be Useful for the Pharmaceutical Industry: Regulatory Attention

There is no need to “advertently promote”, “exaggerate”, or “impose” the pig CARPA test on the pharmaceutical industry or regulatory agencies (Claim 7 in Table 2), as the model has already been noticed and utilized in these spheres. Most notably, it was used in the development of safe administration protocol for nucleic acid-containing solid lipid nanoparticles [37], such as Patisiran (Onpattro), the first FDA approved targeted therapy of a genetic disease based on mRNA interference [80,81]. Numerous other examples are parts of new drug application dossiers (unpublished data).

In general, the question that the pharmaceutical industry needs to balance is the risk/benefit ratio of conducting the pig test. Its potential benefit is the identification of the hazard of a few Grade 4 and 5 SAEs (i.e., anaphylaxis and death) [10], which can halt or stop the commercial development of promising drug candidates in which millions have already been invested. Apart from human tragedies, the regulatory measures entail major press attention with prestige and financial losses for the companies. Recent examples of such events in the nanomedicine field include the PEGylated drugs Peginesatide (Omontys^®^) [82,83], Pegloticase, (Krystexxa^®^) [84,85,86,87], and Pegnivacogin (Revolixys^®^) [88,89,90].

It seems logical that avoiding such calamities by conducting the pig test may far outweigh the risk that a promising drug candidate gets triaged in the preclinical stage based on false positivity in the pig test. In fact, no promising drug candidate needs to be abandoned because the pig assay also enables the testing of the efficacy of preventive and/or therapeutic measures. Previous pig studies have already identified some new approaches to prevent or attenuate CARPA, the PS-NP study [44] being one example. Pretreatment with indomethacin and an anti-C5a antibody [15], desensitization with Doxebo [33], and the design of slow, stepwise infusion protocols [48] represent further options.

Regarding the alarm on “imposing of the pig test on the pharmaceutical industry as a compulsory nanomedicine response test” (Claim 7, Table 2), regulatory agencies have adopted “harmonized standards” (ICH S8 and ICH S6) [91,92] worldwide, which recommend the extension of standard toxicology studies with immune function tests when “the weight-of-evidence” suggests their need. Obviously, a hazard for SAEs does represent such a need, but regulatory agencies generally do not mandate drug developers to follow certain assays over others, nor do they promote or demote any test protocol specifically. Currently, C activation-related toxicity assays, including CARPA, are recommended for consideration in various guidances issued by the US Food and Drug Administration (FDA), European Medicines Agency (EMA), and the World Health Organization (WHO). These guidelines relate to biocompatibility, immune toxicity, and/or bioequivalence [92,93,94,95,96,97], and specifically recommend C and/or CARPA assays in the case of need, such as a risk for infusion reactions to liposome products [96]. The use of pigs for that purpose is in keeping with the increasing use of these animals for toxicity testing as non-rodent alternatives to dogs or non-human primates [98,99], including immune toxicity testing [100]. The porcine CARPA test has been validated in minipigs as well [38], whose benefits in immune toxicology testing is increasingly being recognized [101,102].

It might be an underestimation of the wisdom and vigilance of experts involved in making regulatory recommendations to assume that a misleading model would be made compulsory, or a useful model would be disallowed because of *ex cathedra* judgments on it without sufficient experimental support [50,51].

## 4. The Paradox of Healthy Disease Model

The ambiguities surrounding the human relevance of the pig CARPA test must have a reason, most likely the use of healthy pigs as a disease model. While association of guinea pigs with hypersensitivity tests has a long tradition [103,104,105,106,107], the idea that healthy pigs provide a genetically determined natural model for nanomedicine-induced HSRs may not be the easiest concept to grasp in the vastly multidisciplinary field of nanomedicine. However, there are some unmistakable facts that should distinguish the pig CARPA model from the standard immune toxicity tests run in pigs or minipigs. In the latter case, the tests are done at the therapeutic and higher doses of the drug, while the doses tested in pigs are 2–3 orders of magnitude lower than their therapeutic dose (studies in Table 1), and even much lower than their toxic dose in men or other toxicity models.

## 5. Concordant Symptoms of Pseudoallergy in Pigs and Humans

The above concept on the disease model nature of the porcine CARPA tests was based on the presumption that pigs provide a true model of human nanomedicine-induced HSRs, shown by the similarity of diseases symptoms, technically called “concordance” of symptoms. However, because pigs cannot complain about dyspnea, pain, or anxiety, and man cannot be cannulated for extensive hemodynamic analysis including the measurement of pulmonary arterial pressure, the definition of concordance needs to be extended here to mechanistic concordance, i.e., clinical symptoms taken concordant with experimentally detected physiological changes that explain the clinical symptoms. With this definition, the human symptoms of HSRs, namely dyspnea, chest pain, back pain, tachy- or bradycardia, arrhythmia, light headedness, confusion, fear of death, and panic, developing within minutes after starting the infusion of reactogenic drugs, can be considered as concordant with the circulatory derangement of pigs and minipigs that develop within 2–3 min after injection of reactogenic drugs. The latter derangement, referred to as cardiopulmonary distress, entails transient cardiac, cerebral, and other organ ischemia, which explain the human symptoms. The cutaneous flushing and rash appear identical in man and pigs, as is the pseudo-anaphylactic (cardiac) shock, wherein the tachycardia turns into bradyarrhythmia before death, a known premortal sign in lethal shock in man [24].

As for the concordance of blood cells changes in pigs and man, leukopenia followed by leukocytosis and/or thrombocytopenia were described during drug-induced HSRs in man as C-activation-related [108,109,110,111], just as in pigs [15], rats [58,112], mice [20], and monkeys [113].

Among the non-cellular biomarkers of HSRs, the rise of soluble C terminal complex (sC5b-9) has been shown during HSRs to liposomal doxorubicin (Doxil) in both pigs [47] and cancer patients [114]. Furthermore, the HSR to Doxil follows the same time course and has similar trigger dose in pigs as in humans [25] and the reaction to other reactogenic drugs can be attenuated in pigs by slow infusion [24,48], just as in man [115].

Importantly, not only NPs can cause HSRs that are concordant with physiological changes in the pig model. Kishimoto et al. showed that pigs, unlike rats and other species, provided a good model to recapitulate the heparin-induced HSRs of dialysis patients in the US and Germany during 2007–2008 [29]. The reactions in hundreds of patients, causing the death of near a hundred patients, were characterized by hypotension, shortness of breath, and other typical symptoms of CARPA occurring within 30 min after heparin administration [29,116]. The culprit in these cases was not anti-heparin antibodies, but a contaminant of heparin, namely, oversulfated chondroitin sulfate (OSCS). In parallel with the pseudoallergy symptoms, this linear hetero-polysaccharide caused rises of plasma C5a, C3a, kallikrein, and bradykinin [29,116], indicating the coupling of CARPA with contact system activation.

**Figure 5:**
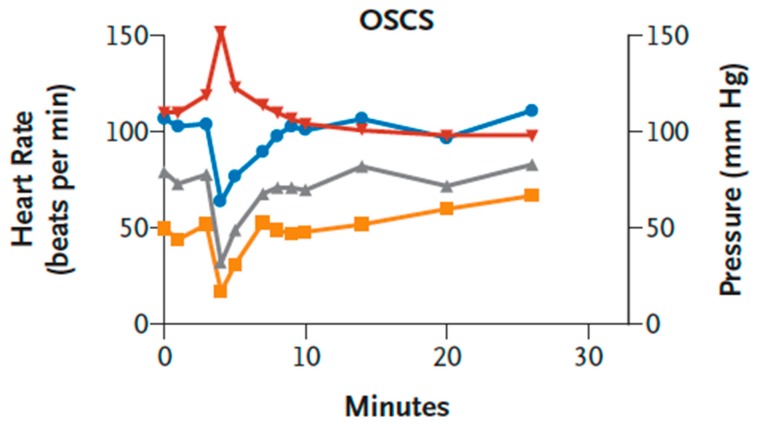
Hemodynamic effects of oversulfated chondroitin sulfate (OSCS) in pigs. Anesthetized Yorkshire crossbred pigs (3–6 pigs per group) were treated with a single intravenous bolus (5 mg per kilogram) of synthetic OSCS. Representative data for the heart rate (red), the mean arterial pressure (gray), the systolic blood pressure (blue), and the diastolic blood pressure (yellow) are shown. Figure reproduced from Ref. [29], with permission.

As shown in Figure 5, the hypotension and tachycardia could be mimicked in pigs—and only in pigs—by i.v. injection of OSCS. Moreover, the reaction proceeded with identical kinetics as seen in pigs injected with C activating NPs (Figure 1, Figure 3 and Figure 4).

A further example for the concordance of immune mechanism and symptoms of NP-induced HSRs in man and pigs can be identified in the “Radar” and “Regulate-PCI” (PCI: percutaneous coronary intervention) trials that tested the efficacy and safety of the PEGylated aptamer anticoagulant, Pegnivacogin (Revolixys kit) [88,89,90]. These trials were stopped because of HSR-related anaphylactoid reactions in a few patients who had high levels of preformed anti-PEG antibodies in their blood [88,89,90]. This mechanism, namely anti-PEG antibody-induced C activation leading to pseudo-anaphylaxis, has been recently reproduced in pigs using PEGylated liposomes [47]. Likewise, in other clinical studies on Pegloticase (Krystexxa), a PEGylated recombinant uricase used for the treatment of refractory gout but later withdrawn from the market because of HSRs, the reactions were shown to be correlated with preexisting and induced anti-PEG Abs and rapid loss of efficacy [84,85,86,87,117]. In these studies, too, the HSRs, as well as the loss of clinical efficacy of the drug, are consistent with CARPA, whereupon the loss of drug efficacy can be explained with the mechanism described in pigs [47], i.e., accelerated blood clearance of C-opsonized, anti-PEG antibody-bound drug. Thus, pigs may provide a model not only for HSRs but also for loss of therapeutic efficacy in the case of certain (PEGylated) drugs.

In addition to the above clinical data attesting to concordance between NP-induced HSRs in pigs and humans, we reported the coincidence of HSRs in pigs with historic data on HSRs in man in the case of low-molecular weight dextran-coated superparamagnetic iron oxide nanoparticles, Sinerem and Resovist [118].

## 6. The Predictive Power of the Pig Test

It needs to be re-emphasized that the reference population to which the prevalence of pig reactions to certain drugs needs to be compared is not the normal human population but the population of patients who are hypersensitive to the same drug or agent. Depending on the drug, this population varies between a broad range of 0.01% and 80%, median values for different drugs roughly being in the 2–10% range. As for the predictive power of the pig test in terms of sensitivity, specificity, positive and negative predictive values, such statistical calculations using, for example 2 × 2 tables [119], can only be performed when sufficient experimental and clinical data are available, which is not the case at present. Statistical calculations of the pig assay’s predictive power are hampered not only by the low occurrence rate of HSRs, but also by the lack of standard protocols of drug administration and anti-allergic premedication in different patients. Thus, even if we had substantially more patient information on HSRs to a drug, their extensive premedication and immediate stopping of the infusion in reacting patients prevent a truly quantitative correlation of symptoms in man and pigs. Thus, the pig assay’s false positivity would be due to medical intervention rather than inappropriateness of the model.

Nevertheless, despite these uncertainties, in absence of alternative approaches of HSR prediction, the discussed concordances give rationale for the use of the pig test to qualitatively assess the reactogenicity of different drugs with the understanding that positivity in the test predicts a general danger for HSRs in hypersensitive patients without quantifying the risk for actual patients or treatment protocols.

## 7. Research Needed to Further Validate the Pig Model

It follows from the above difficulties of correlation analysis between porcine and human HSRs that future studies aimed to further validate the pig model will have to reproduce the human treatment protocol as much as possible, using species-adjusted therapeutic and initial-exposure bolus doses. In addition, the reactions will have to be conducted under identical or similar experimental conditions regarding the pig source and age, and the HSRs will have to be quantified via standardizable methods. Regarding the latter, the studies to date point to PAP as the most reproducible and quantitative measure of HSR. However, it is also shown in Figure 3 that the SAP and heart rate also change as well as the individual blood cell counts, most importantly those of granulocytes and platelets whose changes are not necessarily paralleling. Furthermore, the plasma levels of vasoactive inflammatory mediators (TXB2, PAF, and leukotrienes) also change to different degrees during CARPA. Our attempts in the past to give a combined index for the quantification of porcine CARPA, called cardiopulmonary abnormality score (CAS) [28], embraced all physiological changes that we could measure. However, other scoring methods are also advisable, one being the principal component analysis [120].

## 8. Problems in the Criticism of the Pig Model

This publication was initiated by the vociferous disapproval of the use of pigs as a model for drug-induced HSRs in recent review articles [50,51], conceived after >30 years use of the model in research and preclinical drug development (Table 1). Obviously, shifts of scientific paradigms are essential for progress, for which one should be open, but the attempts in Refs. [50,51] to change the professional recognition and public image of the pig model did not hold up to closer scrutiny. Our analysis points to many inaccuracies and gaps in the critic’s rationale, including linking the HSRs only to PIM-cell derived TXA2; qualifying the dose-dependent, quantitative, and specific physiological changes as “global”; confusing the purpose of the pig assay by mixing up standard toxicity and disease models; misunderstanding regulatory and industrial procedures; and implying commercial ends in the motivation of basic research efforts in the subject. In addition, the authors do not worry about major self-contradictions, most prominently the acknowledgement that human HSRs are “outwardly reproducible in pigs” [44,50,51,59] (which is the ultimate goal of using animals to study human diseases) and the promotion of a new strategy for the prevention of NP-induced HSRs using the same model, which is now being taunted as “inappropriate”, “misleading”, and “scientifically questionable”. The latter high-profile study [44] provided strong experimental evidence for the capability of the porcine CARPA model to distinguish reactogenic from non-reactogenic NPs based on particle geometry, suggesting that rod- and disk-shape PS-NPs are less reactogenic than spherical ones [44]. The question is, therefore, whether this approach of preventing HSRs can be “advertently” promoted further, or the story perhaps needs revisiting as was done [19,45] because of premature postulation of the absence of C activation in the same study [44].

Moghimi et al. stated that “Since, a population of PIMs are believed to be the likely source of thromboxane, and the fact that pulmonary hemodynamic and lymph dynamic changes occur in a dose-dependent fashion to particle injection, *testing of nanomedicine safety in porcine (and other ruminants) will most likely induces cardiopulmonary distress*.” [50] This sentence appears to be a distorted reproduction from the following sentence in Ref. [53]; “Our observations suggest that a population of pulmonary intravascular macrophages is likely to be the source of the thromboxane and the pulmonary hemodynamic and lymph dynamic changes that occur in a dose-dependent fashion, *although interactions between liposomes, leukocytes, or endothelial cells, in addition to the macrophages, have not been completely ruled out.*” Thus, the second (*italicized*) part of the “copy-pasted” sentence was replaced by a logically incoherent self-supporting conclusion *(also italicized)* leaving out an essential portion in the original paper that offered alternatives to the phagocytosis-related TXA2 hypothesis. Likewise, the suggestion in Ref. [53] that “liposomes could conceivably activate production of arachidonic acid metabolites by endothelial cells or the large population of neutrophils in the sheep lung before being phagocytosed by the intravascular macrophages“ has also been neglected. The latter effect, proposed 32 years ago, still represents a likely, yet unexplored explanation for the C-independent “second hit” on PIM and other cells involved in NP-induced HSRs, that may act in synergism with the anaphylatoxin “hit” [16,19].

In light of these deviations from balanced data presentation and judgment, the alarming language “inappropriate”, “misleading”, “scientifically questionable”, and “should not be advertently promoted”, more appropriately characterize the critical authors’ approach and their over-generalization without scientific evidence.

## 9. Conclusions and Future Perspectives

With the advance of complex, targetable nanomedicines (as well as many other biologics and non-biologic complex drugs or NBCDs) that are recognized by the immune system as foreign, the prediction of potential SAEs will have increasing importance in the future to meet the safety mandates of regulatory agencies. The porcine CARPA test may find utility for SAE hazard assessment and mitigation as an extension of standard toxicology protocols on a case by case basis, wherein “the weight-of-evidence” points to a need for HSR risk analysis. The test satisfies the “3R” precondition of a good animal model, namely robustness, reproducibility, and human relevance [121]. Furthermore, it offers a new tool in allergy, circulatory, and toxicology research at their cross-section with nanomedicine. Obviously, in this context, it is essential to ensure that the experimental conditions are set in a clinically relevant manner, the results are correctly interpreted after consideration of additional validation parameters, and that they are integrated into other experimental and clinical data.

Beside advantages, all animal models have certain limitations, and to decide which animal model is ideal to predict human responses to drugs has always been a contentious issue [119,121]. Note that we are not claiming that the porcine CARPA model is the only one, or the best model, to predict HSRs. However, at least the critical issues discussed in this review were clarified as much as our current knowledge enabled.

From our perspective, the pig model’s real challenge for routine safety evaluations lies in the complex logistics, sophisticated instrumentation, and labor intensity involving surgical procedures, the possibility of tachyphylaxis (self-induced tolerance), and the variation of physiological responses to different test drugs and agents. In fact, some or all of these may contribute to making it difficult to standardize the test in terms of drug dose, drug administration protocol, sample collection, and analyte panel in the case of different drugs. These procedures and analyzed variables need to be selected and optimized on a case-by-case basis. However, once this preparative phase is done, the responses are usually highly reproducible in the case of unchanged experimental conditions.

Scientific debates such as the present one on the pig model lead to a better understanding of unclear issues. In the present case, the debate has led to compilation of the experimental use (Table 1) and concordance of the model with human HSR (Section 5) for the first time, as well as to better clarification of the purpose of the model (hazard identification) in preclinical immunotoxicology testing. We believe there is now better justification for recommending the model for pharmaceutical safety testing with or without regulatory mandate. Thus, the rebutted critical reviews [50,51] can be acknowledged as indirectly advancing the effort to make nanomedicines safer.

## Figures and Tables

**Table 1:** Chronological list of pig studies which included the analysis of hemodynamic changes and other endpoints of hypersensitivity reactions to i.v. drugs.

Year	Tested Drugs/Agents *	Major Findings on HSR **	Ref.
1989	Cholesterol-containing liposomes used for immunizing pigs against hypercholesterolemia-induced arteriosclerosis	Liposomes caused major cardiopulmonary distress and TXA2 release via anti-cholesterol antibody-mediated C activation in pig blood.	[21]
1992	Albunex microspheres used as ultrasound contrast agents	HSR involves TXA2-mediated pulmonary hypertension in pigs.	[22]
1994		NPs are cleared mainly by pulmonary intravascular macrophages (PIMs) in the lung of pigs.	[23]
1999	Liposome-encapsulated hemoglobin used for blood substitution and control liposomes	Hemodynamic changes are due to C activation with subsequent secretion of TXA2. PAP is dose-dependent, highly reproducible endpoint of HSRs.	[15]
2000	Different liposomes applied to dissect the structural factors contributing to pulmonary hypertension in pigs	Vesicle size, lamellarity, charge and infusion speed are all critical determinants of the rise of PAP.	[24]
	PEGylated liposomal doxorubicin (Doxil/Caelyx), a reactogenic anticancer drug	Doxil activates C in vitro and its dose-dependent hemodynamic effects in pigs mimic the human HSRs to this drug.	[25]
2005	Negatively charged multilamellar vesicles applied as a model for reactogenic liposomes	The hemodynamic disturbance during HSRs is also manifested in cerebrovascular changes, explaining the psychic symptoms of HSRs.	[26,27]
2006		The symptoms of HSRs reproduce those of cardiac anaphylaxis. The reaction can be reproduced only partially with injection of C5a.	[28]
2008	Oversulfated chondroitin sulfate (OCS), a contaminant of heparin that caused a US nationwide outbreak of severe adverse reactions during 2007–2008	OCS induced contact and complement system activation and cardiopulmonary distress only in pigs but not in other species, mimicking the human symptoms of severe heparin reactions.	[29]
2010	Liposomal bisphosphonates (LBPs) developed for the prevention of myocardial infarction via macrophage inhibition	LBPs triggered no or minor HSRs in pigs, which correlated with their C activating capability in vitro.	[30]
2011	PEI-PEG block-copolymers used as models for polymeric drug carrier nanosystems	25K-PEI activated C in vitro and caused HSRs in pigs; its PEGylation decreased, but did not eliminate these effects.	[31]
2012	PEGylated liposomal doxorubicin (Doxil, Caelyx) and liposomal amphotericin-B (AmBisome), both are reactogenic in patients	Doxil and AmBisome activated C in vitro and caused proportional HSRs in pigs. The effect of Doxil, but not of AmBisome, was tachyphylactic.	[32,33]
	Hemoglobin vesicles (HbVs) used as an oxygen carrier blood substitute	By optimizing the lipid composition of HbVs both C activation and the HSR of pigs could be attenuated. The reaction was tachyphylactic.	[34]
2014	TRO40303, a cardioprotective sterane compound, inhibitor of transitional permeability pores in mitochondria	Consistent with the safety and tolerance in a phase I trial, TRO40303 did not activate C and caused HSR in pigs.	[35]
	Intralipid, used for reversing the symptoms of local anesthetic overdose	Intralipid caused major HSR in pigs, although C activation could not be detected in pig blood in vitro.	[36]
	Inclisiran, a siRNA-containing LNP formulation inhibiting PCSK9 protein to reduce plasma LDL	A stepwise micro-dosing protocol is reaction-free in pigs, suggesting safety in patients.	[37]
2016–2018	Known reactogenic nanomedicines (Doxil, AmBisome, Cremophor EL)	The hemodynamic derangement and other changes caused by reactogenic nanomedicines were similar in pigs and Göttingen miniature pigs.	[38]
	Nano-systems intended for cardiovascular applications (liposomes, LNPs, polymeric and iron oxide NPs)	The non-reactive NPs were suggested to have the least risk for HSRs in man.	[39]
	Nitroglycerin encapsulated in 1,3-diamidophospholipid-containing, shear-responsive liposomes developed to alleviate coronary vasoconstriction	Despite irregular size, these NPs did not activate C and were not reactogenic in pigs.	[40,41]
	A new type of superparamagnetic iron oxide NPs (SPIONdex) used as MRI contrast agents	C activation and HSR in pigs can be eliminated by reducing the size of SPIONdex NPs.	[42,43]
	Polystyrene NPs (PS-NPs) used as a model for reactogenic drug delivery nano-systems	PS-NP-induced cardiopulmonary distress depends on the shape of particles, spheres being more reactogenic than rods or disks. C activation was not measurable in pig whole blood.	[44]
		Spherical PS-NP-induced cardiopulmonary distress in pigs showed significant correlation with C activation in human serum. PS-NPs were opsonized in pig serum by C3 derivatives, indicating C activation.	[45]
	Hemostatic NPs based on PEG-PLGA-PLL-PEG-cRGD copolymers, developed to control traumatic blood loss	In a porcine liver injury model, these NPs led to massive vasodilation and exsanguination due to CARPA. This adverse effect could be attenuated by tailoring the zeta potential of NPs.	[46]
2019	Doxil and placebo Doxil (Doxebo) used to clarify the mechanism of HSRs to Doxil and other PEGylated NPs	C activation and the HSR caused by Doxil was greatly amplified in Doxebo-immunized animals in which the anti-PEG IgM levels were increased. This provides evidence for the causal role of classical pathway C activation in Doxil reactions.	[47]
	Liposomal cortisol phosphate developed against chronic inflammatory diseases	Consistent with the human practice, slow, stepwise infusion with micro-dosing minimizes the risk for HSRs.	[48]
	TC^99m^-Fucoidan, a sulfated fucose-rich polysaccharide developed for the detection of P-selectin expression in cardiovascular diseases	The drug did not cause C activation or HSR in pigs, suggesting safety for human use for the imaging of activated endothelium.	[49]

* Trade names and abbreviations: AmBisome, liposomal amphotericin-B; C, complement; Doxil, PEGylated liposomal doxorubicin; HSR, hypersensitivity reaction; HbVs, hemoglobin vesicles; LEH, liposome-encapsulated hemoglobin; LNPs, lipid nanoparticles; LDL, low density lipoprotein; NPs, nanoparticles; OCS, oversulfated chondroitin sulfate; PEI, polyethylene-imine; PEG-PLGA-PLL-PEG-cRGD, cyclic peptide (arginine-glycine-aspartic-glutamic-valine acid, cRGD)-modified monomethoxy (polyethylene glycol)-poly (d,l-lactide-*co*-glycolide)-poly (l-lysine) nanoparticles; siRNA, small inhibitory ribonucleic acid; PS-NPs, polystyrene NPs; SPIONs, superparamagnetic iron oxide nanoparticles; TRO40303, 3,5-seco-4-nor-cholestan-5-one oxime-3-ol-containing liposomes; PCSK9, proprotein convertase subtilisin/kexin type 9 (LDL uptake blocker). ** Conclusions on hemodynamic, TXA2, and other physiological changes observed in response to i.v. administration of test agents.

**Table 2:** Facts and arguable conclusions regarding the pig model of infusion reactions *.

Experimental Facts:	References
1. PIM cells are abundantly present in the lung of cloven-hoofed members of the mammalian order Artiodactyla, including pigs, sheep, goats, cattle, horse, etc.	[50,51,52,53,54,55,56]
2. PIM cells are highly phagocytotic and can secret, among others, vasoactive eicosanoids, including thromboxane A_2_ (TXA2).	
3. The vasoactivity of TXA2 is a key contributor to the massive hemodynamic changes following NP injection of pigs and other animals.	[15,20,48,50,51,52,53,54,55,56,57,58]
**Arguable Claims:**	
4. The NP-induced hemodynamic changes in pigs are due to robust phagocytosis of NPs by PIM cells, the source of thromboxane. *That is, PIM phagocytosis is causally involved in HSR, rather than C activation with stimulation of a variety of cells for proinflammatory response* via *other pathways*.	[44,50,51,59]
5. The hemodynamic response to i.v. nanoparticles is a “global outcome”, *implying omnipresent, uniform, non-specific, non-quantitative cardiovascular changes.*	[50,51].
6. The discordant prevalence of HSRs in pigs and healthy man makes the model irrelevant to humans, excluding otherwise promising nanopharmaceuticals from the development pipeline on safety grounds that are not relevant to wider human populations. *That is, because the HSRs are rare in humans but always observed in the pig model, the pig model overestimates the risk of human HSRs*.	[50,51,60]
7. The pig assay is being advertently promoted and their applications exaggerated or imposed on the pharmaceutical industry as a compulsory nanomedicine response test. *This is a baseless presumption.*	[50,51]

* Italicized text represents explanation to help understanding.

## References

[1] Sear J., Prys-Roberts C. (1983). Hypersensitivity reactions to infusions of Althesin. Anaesthesia.

[2] Pichler W.J. (2006). Adverse side-effects to biological agents. Allergy.

[3] Lenz H.-J. (2007). Management and Preparedness for Infusion and Hypersensitivity Reactions. Oncologist.

[4] Vogel W.H. (2010). Infusion reactions: Diagnosing, Asessment and management. Clin. J. Oncol. Nurs..

[5] Maggi E., Vultaggio A., Matucci A. (2011). Acute infusion reactions induced by monoclonal antibody therapy. Expert Rev. Clin. Immunol..

[6] Szebeni J., Simberg D., Gonzalez-Fernandez A., Barenholz Y., Dobrovolskaia M.A. (2018). Roadmap and strategy for overcoming infusion reactions to nanomedicines. Nat. Nanotechnol..

[7] Szebeni J., Bawa R., Bawa R., Szebeni J., Webster T.J., Audette G.F. (2019). Immunological Issues with Medicines of Nano Size: The Price of Dimension Paradox. Immune Aspects of Biopharmaceuticals and Nanomedicines.

[8] Trotti A., Colevas A., Setser A., Rusch V., Jaques D., Budach V., Langer C., Murphy B., Cumberlin R., Coleman C. (2003). CTCAE v3.0: Development of a comprehensive grading system for the adverse effects of cancer treatment. Semin. Radiat. Oncol..

[9] Tang A.W. (2003). A practical guide to anaphylaxis. Am. Fam. Physician.

[10] National Cancer Institute Common Terminology Criteria for Adverse Events (CTCAE). https://ctep.cancer.gov/protocolDevelopment/electronic_applications/ctc.htm.

[11] Sahiner U.M., Yavuz S.T., Gokce M., Buyuktiryaki B., Altan I., Aytac S., Tuncer M., Tuncer A., Sackesen C. (2013). Anaphylactic reaction to polyethylene-glycol conjugated-asparaginase: Premedication and desensitization may not be sufficient. Pediatr. Int..

[12] Fumery M., Tilmant M., Yzet C., Brazier F., Loreau J., Turpin J., Le Mouel J.P., Goeb V., Nguyen-Khac E., Singh S. (2019). Premedication as primary prophylaxis does not influence the risk of acute infliximab infusion reactions in immune-mediated inflammatory diseases: A systematic review and meta-analysis. Dig. Liver Dis..

[13] Madrigal-Burgaleta R., Bernal-Rubio L., Berges-Gimeno M.P., Carpio-Escalona L.V., Gehlhaar P., Alvarez-Cuesta E. (2019). A Large Single-Hospital Experience Using Drug Provocation Testing and Rapid Drug Desensitization in Hypersensitivity to Antineoplastic and Biological Agents. J. Allergy Clin. Immunol. Pract..

[14] Szebeni J. (2014). Complement activation-related pseudoallergy: A stress reaction in blood triggered by nanomedicines and biologicals. Mol. Immunol..

[15] Szebeni J., Fontana J.L., Wassef N.M., Mongan P.D., Morse D.S., Dobbins D.E., Stahl G.L., Bünger R., Alving C.R. (1999). Hemodynamic changes induced by liposomes and liposome-encapsulated hemoglobin in pigs: A model for pseudoallergic cardiopulmonary reactions to liposomes. Role of complement and inhibition by soluble CR1 and anti-C5a antibody. Circulation.

[16] Szebeni J., Bedocs P., Csukás D., Rosivall L., Bünger R., Urbanics R. (2012). A porcine model of complement-mediated infusion reactions to drug carrier nanosystems and other medicines. Adv. Drug Deliv. Rev..

[17] Urbanics R., Bedocs P., Szebeni J. (2015). Lessons learned from the porcine CARPA model: Constant and variable responses to different nanomedicines and administration protocols. Eur. J. Nanomed..

[18] Szebeni J., Bedocs P., Dézsi L., Urbanics R. (2018). A porcine model of complement activation-related pseudoallergy to nano-pharmaceuticals: Pros and cons of translation to a preclinical safety test. Precis. Nanomed..

[19] Szebeni J. (2018). Mechanism of nanoparticle-induced hypersensitivity in pigs: Complement or not complement?. Drug Discov. Today.

[20] Őrfi E., Mészáros T., Hennies M., Fülöp T., Dézsi L., Nardocci A., Rosivall L., Hamar P., Neun B.W., Dobrovolskaia M.A. (2019). Acute physiological changes caused by complement activators and amphotericin B-containing liposomes in mice. Int. J. Nanomed..

[21] Wassef N.M., Johnson S.H., Graeber G.M., Swartz G.M., Schultz C.L., Hailey J.R., Johnson A.J., Taylor D.G., Ridgway R.L., Alving C.R. (1989). Anaphylactoid reactions mediated by autoantibodies to cholesterol in miniature pigs. J. Immunol..

[22] Øistensen J., Hede R., Myreng Y., Ege T., Holtz E. (1992). Intravenous injection of AlbunexRmicrospheres causes thromboxane mediated pulmonary hypertension in pigs, but not in monkeys or rabbits. Acta Physiol. Scand..

[23] Walday P., Tolleshaug H., Gjøen T., Kindberg G.M., Berg T., Skotland T., Holtz E. (1994). Biodistributions of air-filled albumin microspheres in rats and pigs. Biochem. J..

[24] Szebeni J., Baranyi L., Savay S., Bodo M., Morse D.S., Basta M., Stahl G.L., Bünger R., Alving C.R. (2000). Liposome-induced pulmonary hypertension: Properties and mechanism of a complement-mediated pseudoallergic reaction. Am. J. Physiol. Circ. Physiol..

[25] Szebeni J., Baranyi L., Savay S., Lutz H.U., Jelezarova E., Bünger R., Alving C.R. (2000). The Role of Complement Activation in Hypersensitivity to Pegylated Liposomal Doxorubicin (Doxil^®^). J. Liposome Res..

[26] Bodo J.S.M. (2005). Rheoencephalographic evidence of complement activation-related cerebrovascular changes in pigs. J. Cereb. Blood Flow Metab..

[27] Bodo M., Szebeni J., Baranyi L., Savay S., Pearce F.J., Alving C.R., Bünger R. (2005). Cerebrovascular involvement in liposome-induced cardiopulmonary distress in pigs. J. Liposome Res..

[28] Szebeni J., Baranyi L., Savay S., Bodo M., Milosevits J., Alving C.R., Bünger R. (2006). Complement activation-related cardiac anaphylaxis in pigs: Role of C5a anaphylatoxin and adenosine in liposome-induced abnormalities in ECG and heart function. Am. J. Physiol. Circ. Physiol..

[29] Kishimoto T.K., Viswanathan K., Ganguly T., Elankumaran S., Smith S., Pelzer K., Lansing J., Sriranganathan N., Zhao G., Galcheva-Gargova Z. (2008). Contaminated heparin associated with adverse clinical events and activation of the contact system. N. Engl. J. Med..

[30] Epstein-Barash H., Gutman D., Markovsky E., Mishan-Eisenberg G., Koroukhov N., Szebeni J., Golomb G. (2010). Physicochemical parameters affecting liposomal bisphosphonates bioactivity for restenosis therapy: Internalization, cell inhibition, activation of cytokines and complement, and mechanism of cell death. J. Control. Release.

[31] Merkel O.M., Urbanics R., Bedocs P., Rozsnyay Z., Rosivall L., Tóth M., Kissel T., Szebeni J. (2011). In vitro and in vivo complement activation and related anaphylactic effects associated with polyethylenimine and polyethylenimine-graft-poly(ethylene glycol) block copolymers. Biomaterials.

[32] Szebeni J., Bedocs P., Rozsnyay Z., Weiszhár Z., Urbanics R., Rosivall L., Cohen R., Garbuzenko O., Bathori G., Tóth M. (2012). Liposome-induced complement activation and related cardiopulmonary distress in pigs: Factors promoting reactogenicity of Doxil and AmBisome. Nanomedicine.

[33] Szebeni J., Bedocs P., Urbanics R., Bünger R., Rosivall L., Tóth M., Barenholz Y. (2012). Prevention of infusion reactions to PEGylated liposomal doxorubicin via tachyphylaxis induction by placebo vesicles: A porcine model. J. Control. Release.

[34] Sakai H., Suzuki Y., Sou K., Kano M. (2012). Cardiopulmonary hemodynamic responses to the small injection of hemoglobin vesicles (artificial oxygen carriers) in miniature pigs. J. Biomed. Mater. Res. Part A.

[35] Le Lamer S., Paradis S., Rahmouni H., Chaimbault C., Michaud M., Culcasi M., Afxantidis J., Latreille M., Berna P., Berdeaux A. (2014). Translation of TRO40303 from myocardial infarction models to demonstration of safety and tolerance in a randomized Phase I trial. J. Transl. Med..

[36] Bedocs P., Capacchione J., Potts L., Chugani R., Weiszhár Z., Szebeni J., Buckenmaier C.C. (2014). Hypersensitivity Reactions to Intravenous Lipid Emulsion in Swine. Anesthesia Analg..

[37] Kasperovic P., Gollob J. (2014). Dosages and Methods for Delivering Lipid Formulated Nucleic Acid Molecules; PCT No. PCT/US2014/036915.

[38] Jackman J.A., Mészáros T., Fülöp T., Urbanics R., Szebeni J., Cho N.-J. (2016). Comparison of complement activation-related pseudoallergy in miniature and domestic pigs: Foundation of a validatable immune toxicity model. Nanomedicine.

[39] Matuszak J., Baumgartner J., Zaloga J., Juenet M., Da Silva A.E., Franke D., Almer G., Texier I., Faivre D., Metselaar J.M. (2016). Nanoparticles for intravascular applications: Physicochemical characterization and cytotoxicity testing. Nanomedicine.

[40] Bugna S., Buscema M., Matviykiv S., Urbanics R., Weinberger A., Meszaros T., Szebeni J., Zumbuehl A., Saxer T., Müller B. (2016). Surprising lack of liposome-induced complement activation by artificial 1,3-diamidophospholipids in vitro. Nanomedicine.

[41] Buscema M., Matviykiv S., Meszaros T., Gerganova G., Weinberger A., Mettal U., Mueller D., Neuhaus F., Stalder E., Ishikawa T. (2017). Immunological response to nitroglycerin-loaded shear-responsive liposomes in vitro and in vivo. J. Control. Release.

[42] Unterweger H., Janko C., Schwarz M., Dézsi L., Urbanics R., Matuszak J., Őrfi E., Fülöp T., Bäuerle T., Szebeni J. (2017). Non-immunogenic dextran-coated superparamagnetic iron oxide nanoparticles: A biocompatible, size-tunable contrast agent for magnetic resonance imaging. Int. J. Nanomed..

[43] Unterweger H., Dezsi L., Matuszak J., Janko C., Pöttler M., Jordan J., Bäuerle T., Szebeni J., Fey T., Boccaccini A.R. (2018). Dextran-coated superparamagnetic iron oxide nanoparticles for magnetic resonance imaging: Evaluation of size-dependent imaging properties, storage stability and safety. Int. J. Nanomed..

[44] Wibroe P.P., Anselmo A.C., Nilsson P., Sarode A., Gupta V., Urbanics R., Szebeni J., Hunter A., Mitragotri S., Mollnes T.E. (2017). Bypassing adverse injection reactions to nanoparticles through shape modification and attachment to erythrocytes. Nat. Nanotechnol..

[45] Mészáros T., Kozma G.T., Shimizu T., Miyahara K., Turjeman K., Ishida T., Barenholz Y., Urbanics R., Szebeni J. (2018). Involvement of complement activation in the pulmonary vasoactivity of polystyrene nanoparticles in pigs: Unique surface properties underlying alternative pathway activation and instant opsonization. Int. J. Nanomed..

[46] Onwukwe C., Maisha N., Holland M., Varley M., Groynom R., Hickman D., Uppal N., Shoffstall A., Ustin J., Lavik E.B. (2018). Engineering Intravenously Administered Nanoparticles to Reduce Infusion Reaction and Stop Bleeding in a Large Animal Model of Trauma. Bioconjugate Chem..

[47] Kozma G.T., Mészáros T., Vashegyi I., Fülöp T., Örfi E., Dézsi L., Rosivall L., Bavli Y., Urbanics R., Mollnes T.E. (2019). Pseudo-anaphylaxis to Polyethylene Glycol (PEG)-Coated Liposomes: Roles of Anti-PEG IgM and Complement Activation in a Porcine Model of Human Infusion Reactions. ACS Nano.

[48] Fülöp T., Kozma G.T., Vashegyi I., Mészáros T., Rosivall L., Urbanics R., Storm G., Metselaar J.M., Szebeni J. (2019). Liposome-induced hypersensitivity reactions: Risk reduction by design of safe infusion protocols in pigs. J. Control. Release.

[49] Chauvierre C., Aid-Launais R., Aerts J., Chaubet F., Maire M., Chollet L., Rolland L., Bonafé R., Rossi S., Bussi S. (2019). Pharmaceutical Development and Safety Evaluation of a GMP-Grade Fucoidan for Molecular Diagnosis of Cardiovascular Diseases. Mar. Drugs.

[50] Moghimi S.M., Simberg D. (2018). Translational gaps in animal models of human infusion reactions to nanomedicines. Nanomedicine.

[51] Moghimi S.M., Simberg D., Skotland T., Yaghmur A., Hunter A.C., Hunter C. (2019). The Interplay Between Blood Proteins, Complement, and Macrophages on Nanomedicine Performance and Responses. J. Pharmacol. Exp. Ther..

[52] Warner A.E., Brain J.D. (1986). Intravascular pulmonary macrophages: A novel cell removes particles from blood. Am. J. Physiol. Integr. Comp. Physiol..

[53] Miyamoto K., Schultz E., Heath T., Mitchell P.M.D., Albertine K.H., Staub N.C. (1988). Pulmonary intravascular macrophages and hemodynamic effects of liposomes in sheep. J. Appl. Physiol..

[54] Schneberger D., Aharonson-Raz K., Singh B. (2012). Pulmonary intravascular macrophages and lung health: What are we missing?. Am. J. Physiol. Cell. Mol. Physiol..

[55] Nakano T., Miyamoto K., Nishimura M., Aida A., Aoi K., Kawakami Y. (1994). Role of pulmonary intravascular macrophages in anti-platelet serum-induced pulmonary hypertension in sheep. Respir. Physiol..

[56] Csukás D., Urbanics R., Wéber G., Rosivall L., Szebeni J. (2015). Pulmonary intravascular macrophages: Prime suspects as cellular mediators of porcine CARPA. Eur. J. Nanomed..

[57] Szebeni J., Spielberg H., Cliff R.O., Wassef N.M., Rudolph A.S., Alving C.R. (1997). Complement activation and thromboxane A2 secretion in rats following administration of liposome-encapsulated hemoglobin: Inhibition by soluble complement receptor type 1. Art Cells Blood Subs. Immob. Biotechnol..

[58] Dézsi L., Fülöp T., Mészáros T., Szénási G., Urbanics R., Vázsonyi C., Őrfi E., Rosivall L., Nemes R., Kok R.J. (2014). Features of complement activation-related pseudoallergy to liposomes with different surface charge and PEGylation: Comparison of the porcine and rat responses. J. Control. Release.

[59] Moghimi S.M. (2018). Nanomedicine safety in preclinical and clinical development: Focus on idiosyncratic injection/infusion reactions. Drug Discov. Today.

[60] Skotland T. (2017). Injection of nanoparticles into clover-hoof animals: Asking for trouble. Theranostics.

[61] Hänsch G.M., Seitz M., Martinotti G., Betz M., Rauterberg E.W., Gemsa D. (1984). Macrophages release arachidonic acid, prostaglandin E2, and thromboxane in response to late complement components. J. Immunol..

[62] Lefer A.M., Smith J.B., Nicolaou K.C., Kovach A.G.B., Hamar J., Szabo L. (1981). Cardiovascular actions of two thromboxane A2 analogs. Cardiovascular Physiology Microcirculation and Capillary Exchange; Proceedings of the 28^th^ Congress of Physiological Sciences, Budapest, 1980.

[63] Nakahata N. (2008). Thromboxane A2: Physiology/pathophysiology, cellular signal transduction and pharmacology. Pharmacol. Ther..

[64] Rucker D., Dhamoon A.S. Physiology, Thromboxane A2. https://www.ncbi.nlm.nih.gov/books/NBK539817/.

[65] Stahl G.L., Morse D.S., Martin S.L. (1997). Eicosanoid production from porcine neutrophils and platelets: Differential production with various agonists. Am. J. Physiol. Content.

[66] Patko Z., Szebeni J. (2015). Blood cell changes in complement activation-related pseudoallergy. Eur. J. Nanomed..

[67] Kreisel D., Nava R.G., Li W., Zinselmeyer B., Wang B., Lai J., Pless R., Gelman A.E., Krupnick A.S., Miller M.J. (2010). In vivo two-photon imaging reveals monocyte-dependent neutrophil extravasation during pulmonary inflammation. Proc. Natl. Acad. Sci. USA.

[68] Feith G.W., Bogman M.J., Assmann K.J., Van Gompel A.P., Schalkwijk J., Van Rooijen N., Koene R.A. (1997). Decreased PMN accumulation and glomerular damage by clodronate liposome treatment in PMN-dependent anti-GBM nephritis in mice. Exp. Nephrol..

[69] Albunex Side Effects. https://wwweverydayhealthcom/drugs/albunex.

[70] Geny B., Mettauer B., Muan B., Bischoff P., Epailly E., Piquard F., Eisenmann B., Haberey P. (1993). Safety and efficacy of a new transpulmonary echo contrast agent in echocardiographic studies in patients. J. Am. Coll. Cardiol..

[71] De Groot M., Van Zwieten-Boot B.J., Van Grootheest A.C. (2004). [Severe adverse reactions after the use of sulphur hexafluoride (SonoVue) as an ultrasonographic contrast agent]. Ned. Tijdschr. Geneeskd..

[72] Yamaya Y., Niizeki K., Kim J., Entin P., Wagner H., Wagner P.D. (2004). Anaphylactoid response to Optison(R) and its effects on pulmonary function in two dogs. J. Vet. Med Sci..

[73] Aggeli C., Giannopoulos G., Roussakis G., Christoforatou E., Marinos G., Toli C., Pitsavos C., Stefanadis C. (2008). Safety of myocardial flash-contrast echocardiography in combination with dobutamine stress testing for the detection of ischaemia in 5250 studies. Heart.

[74] Wei K., Mulvagh S.L., Carson L., Davidoff R., Gabriel R., Grimm R.A., Wilson S., Fane L., Herzog C.A., Zoghbi W.A. (2008). The safety of deFinity and Optison for ultrasound image enhancement: A retrospective analysis of 78,383 administered contrast doses. J. Am. Soc. Echocardiogr..

[75] Geleijnse M.L., Nemes A., Vletter W.B., Michels M., Soliman O., Caliskan K., Galema T.W., Cate F.T. (2009). Adverse reactions after the use of sulphur hexafluoride (SonoVue) echo contrast agent. J. Cardiovasc. Med..

[76] Ionescu A. (2009). Bubble trouble: Anaphylactic shock, threatened myocardial infarction, and transient renal failure after intravenous echo contrast for left ventricular cavity opacification preceding dobutamine stress echo. Eur. J. Echocardiogr..

[77] Solivetti F., Elia F., Musicco F., Bonagura A., Di Leo N., Iera J., Drudi F.M. (2012). Anaphylactic Shock Induced by Sulphur Hexafluoride in an Individual with no History of Heart Disease: Case Report and Literature review. Ultraschall Med..

[78] Levano J.A., Jimenez M.A., Laiseca A., Vives R. (2012). Anaphylactic shock due to SonoVue. Ann. Allergy Asthma Immunol..

[79] Coudray S., Fabre C., Aichoun I., Perez-Martin A. (2017). Anaphylactic shock with an ultrasound contrast agent. J. Med. Vasc..

[80] Hoy S.M. (2018). Patisiran: First Global Approval. Drugs.

[81] Kristen A.V., Ajroud-Driss S., Conceição I., Gorevic P., Kyriakides T., Obici L. (2019). Patisiran, an RNAi therapeutic for the treatment of hereditary transthyretin-mediated amyloidosis. Neurodegener. Dis. Manag..

[82] Bennett C.L., Jacob S., Hymes J., Usvyat L.A., Maddux F.W. (2014). Anaphylaxis and hypotension after administration of peginesatide. N. Engl. J. Med..

[83] (2013). Withdrawal Assessment Report for Omontys. https://wwwemaeuropaeu/en/documents/withdrawal-report/withdrawal-assessment-report-omontys_enpdf.

[84] Calabrese L.H., Kavanaugh A., Yeo A.E., Lipsky P.E. (2017). Frequency, distribution and immunologic nature of infusion reactions in subjects receiving pegloticase for chronic refractory gout. Arthritis Res. Ther..

[85] Hershfield M.S., Ganson N.J., Kelly S.J., Scarlett E.L., Jaggers D.A., Sundy J.S. (2014). Induced and pre-existing anti-polyethylene glycol antibody in a trial of every 3-week dosing of pegloticase for refractory gout, including in organ transplant recipients. Arthritis Res. Ther..

[86] KRYSTEXXA® (Pegloticase Injection), for Intravenous Infusion: Prescribing Information Revised 2018. https://www.accessdata.fda.gov/drugsatfda_docs/label/2012/125293s040lbl.pdf.

[87] (2017). Pegloticase: Withdrawal of its European marketing authorisation is welcome. Prescrire Int..

[88] Ganson N.J., Povsic T.J., Sullenger B.A., Alexander J.H., Zelenkofske S.L., Sailstad J.M., Rusconi C.P., Hershfield M.S. (2015). Pre-existing anti-polyethylene glycol antibody linked to first-exposure allergic reactions to pegnivacogin, a PEGylated RNA aptamer. J. Allergy Clin. Immunol..

[89] Povsic T.J., Lawrence M.G., Lincoff A.M., Mehran R., Rusconi C.P., Zelenkofske S.L., Huang Z., Sailstad J., Armstrong P.W., Steg P.G. (2016). Pre-existing anti-PEG antibodies are associated with severe immediate allergic reactions to pegnivacogin, a PEGylated aptamer. J. Allergy Clin. Immunol..

[90] Povsic T.J., Vavalle J.P., Aberle L.H., Kasprzak J.D., Cohen M.G., Mehran R., Bode C., Buller C.E., Montalescot G., Cornel J.H. (2012). A Phase 2, randomized, partially blinded, active-controlled study assessing the efficacy and safety of variable anticoagulation reversal using the REG1 system in patients with acute coronary syndromes: Results of the RADAR trial. Eur. Heart J..

[91] Food and Drug Administration (FDA) (2006). International Conference on Harmonisation; Guidance on S8 Immunotoxicity Studies for Human Pharmaceuticals; availability. Notice. Fed. Regist..

[92] Food and Drug Administration (FDA) (2012). International Conference on Harmonisation; addendum to International Conference on Harmonisation Guidance on S6 Preclinical Safety Evaluation of Biotechnology-Derived Pharmaceuticals; availability. Notice. Fed. Regist..

[93] Guidance for Industry: Immunotoxicology Evaluation of Investigational New Drugs. https://www.fda.gov/regulatory-information/search-fda-guidance-documents/immunotoxicology-evaluation-investigational-new-drugs.

[94] Hastings K. (2002). Implications of the new FDA/CDER immunotoxicology guidance for drugs. Int. Immunopharmacol..

[95] Association for the Advancement of Medical Instrumentation, International Organization for Standardization (2009). Biological Evaluation of Medical Devices—Part 4: Selection of Tests for Interaction with Blood. ANSI/AAMI/ISO 10993-4:2002/(R).

[96] (2013). Reflection Paper on the Data Requirements for Intravenous Liposomal Products Developed with Reference to an Innovator Liposomal Product. https://www.ema.europa.eu/en/documents/scientific-guideline/reflection-paper-data-requirements-intravenous-liposomal-products-developed-reference-innovator_en-0.pdf.

[97] (2013). Guidelines on the Quality, Safety, and Efficacy of Biotherapeutic Protein Products Prepared by Recombinant DNA Technology. https://www.who.int/biologicals/biotherapeutics/rDNA_DB_final_19_Nov_2013.pdf.

[98] Swindle M.M. (2012). The development of swine models in drug discovery and development. Future Med. Chem..

[99] Swindle M.M., Makin A., Herron A.J., Clubb F.J., Frazier K.S. (2011). Swine as Models in Biomedical Research and Toxicology Testing. Vet. Pathol..

[100] Gerner W., Saalmuller A. (2016). The Immune System of Swine. Encyclopedia of Immunobiology.

[101] Van Mierlo G.J.D., Kuper C.F., de Zeeuw-Brouwer M.L., Schijf M.A., Bruijntjes J.P., Otto M., Ganderup N.C., Penninks A.H. (2013). A sub acute immunotoxicity study in Göttingen minipigs with the immunosuppressive compounds cyclosporin A and dexamethasone. J. Clin. Exp. Pharm..

[102] Peachee V.L., Smith M.J., Beck M.J., Stump D.G., White K.L. (2013). Characterization of the T-dependent antibody response (TDAR) to keyhole limpet hemocyanin (KLH) in the Göttingen minipig. J. Immunotoxicol..

[103] Hicks R., Skeldon N. (1970). The Influence of Adjuvants on Antibody Production and Anaphylactic Hypersensitivity in the Guinea Pig. Int. Arch. Allergy Immunol..

[104] Kostiala A.A. (1971). Delayed hypersensitivity in the guinea pig immunized with killed tubercle bacilli in adjuvant. 1. Development of peritoneal cell migration inhibition, skin reactions and antibodies to tuberculin purified protein derivative. Acta Pathol. Microbiol. Scand. B Microbiol. Immunol..

[105] Verdier F., Chazal I., Descotes J. (1994). Anaphylaxis models in the guinea-pig. Toxicology.

[106] Weaver J.L., Staten D., Swann J., Armstrong G., Bates M., Hastings K.L. (2003). Detection of systemic hypersensitivity to drugs using standard guinea pig assays. Toxicology.

[107] Ricciardolo F.L., Nijkamp F., Rose V., Folkerts G. (2008). The Guinea Pig as an Animal Model for Asthma. Curr. Drug Targets.

[108] Chan C.K., Jarrett F., Moylan J.A. (1976). Acute leukopenia as an allergic reaction to silver sulfadiazine in burn patients. J. Trauma.

[109] Frangi D., Gardinali M., Conciato L., Cafaro C., Pozzoni L., Agostoni A. (1994). Abrupt complement activation and transient neutropenia in patients with acute myocardial infarction treated with streptokinase. Circulation.

[110] Yeh Y.-W., Wang T.-Y., Huang C.-C., Chen Y.-C. (2008). Late-onset hypersensitivity reaction with leukopenia and thrombocytopenia induced by oxcarbazepine treatment in a patient with schizoaffective disorder. J. Clin. Psychiatry.

[111] Michelmann I., Bockmann D., Nurnberger W., Eckhof-Donovan S., Burdach S., Gobel U. (1997). Thrombocytopenia and complement activation under recombinant TNF alpha/IFN gamma therapy in man. Ann Hematol..

[112] Dézsi L., Mészáros T., Őrfi E., Fülöp T., Hennies M., Rosivall L., Hamar P., Szebeni J., Szénási G. (2019). Complement Activation-Related Pathophysiological Changes in Anesthetized Rats: Activator-Dependent Variations of Symptoms and Mediators of Pseudoallergy. Molecules.

[113] Galbraith W.M., Hobson W.C., Giclas P.C., Schechter P.J., Agrawal S. (1994). Complement Activation and Hemodynamic Changes Following Intravenous Administration of Phosphorothioate Oligonucleotides in the Monkey. Antisense Res. Dev..

[114] Chanan-Khan A., Szebeni J., Savay S., Liebes L., Rafique N.M., Alving C.R., Muggia F.M. (2003). Complement activation following first exposure to pegylated liposomal doxorubicin (Doxil^®^): Possible role in hypersensitivity reactions. Ann. Oncol..

[115] Szebeni J., Muggia F., Gabizon A., Barenholz Y. (2011). Activation of complement by therapeutic liposomes and other lipid excipient-based therapeutic products: Prediction and prevention. Adv. Drug Deliv. Rev..

[116] Blossom D.B., Kallen A.J., Patel P.R., Elward A., Robinson L., Gao G., Langer R., Perkins K.M., Jaeger J.L., Kurkjian K.M. (2008). Outbreak of adverse reactions associated with contaminated heparin. N. Engl. J. Med..

[117] Sundy J., Becker M.A., Baraf H.S.B., Barkhuizen A., Moreland L.W., Huang W., Waltrip R.W., Maroli A.N., Horowitz Z. (2008). Pegloticase Phase 2 Study Investigators Reduction of plasma urate levels following treatment with multiple doses of pegloticase (polyethylene glycol-conjugated uricase) in patients with treatment-failure gout: Results of a phase II randomized study. Arthritis Rheum..

[118] Fülöp T., Nemes R., Mészáros T., Urbanics R., Kok R.J., Jackman J.A., Cho N.-J., Storm G., Szebeni J. (2017). Complement activation in vitro and reactogenicity of low-molecular weight dextran-coated SPIONs in the pig CARPA model: Correlation with physicochemical features and clinical information. J. Control. Release.

[119] Shanks N., Greek R., Greek J. (2009). Are animal models predictive for humans?. Philos. Ethic Humanit. Med..

[120] Giuliani A. (2017). The application of principal component analysis to drug discovery and biomedical data. Drug Discov Today..

[121] Everitt J.I. (2015). The future of preclinical animal models in pharmaceutical discovery and development: A need to bring in cerebro to the in vivo discussions. Toxicol. Pathol..

